# Unusual Bladder Metastasis from a Primary Gastric Carcinoma: Two Case Reports and Review of Literature

**DOI:** 10.1155/2020/8848841

**Published:** 2020-11-12

**Authors:** Mohamed Ali Nouioui, Ahmed Saadi, Marouene Chakroun, Amine Oueslati, Meriem Ksentini, Abderrazak Bouzouita, Amine Derouiche, Mohamed Riadh Ben Slama, Soumaya Rammeh, Haroun Ayed, Mohamed Chebil

**Affiliations:** ^1^Department of Urology, Charles Nicolle Hospital of Tunis, University of Tunis El Manar, Tunisia; ^2^Department of Pathology, Charles Nicolle Hospital of Tunis, University of Tunis El Manar, Tunisia

## Abstract

Primary bladder cancer is a frequent malignancy in the urology field, whereas secondary bladder neoplasms from a distant organ are extremely rare. This paper aims to report two rare cases of a secondary tumor of the urinary bladder from a primary gastric tumor and to perform a literature review of similar reported cases in order to better characterize its clinicopathological features and diagnosis in effort to shed light on this rare condition. The final diagnosis of secondary adenocarcinoma was made histologically after transurethral biopsy or resection of the bladder lesion. In one case, the bladder metastasis was a synchronous metastasis, and in the second case, it occurred under chemotherapy five months after initial diagnosis with gastric adenocarcinoma. Secondary adenocarcinoma of the bladder is extremely rare but should be considered when evaluating a bladder lesion in a patient treated for gastric cancer or presenting with gastric symptoms.

## 1. Introduction

Primary bladder cancer is a frequent malignancy in the urological field whereas secondary bladder tumor from distant primary foci is considered an extremely rare condition accounting for 2% from total vesical tumors [1].

Histologically, 54% of those secondary neoplasms of the bladder are adenocarcinomas of which 4.3% are originated from a primary gastric adenocarcinoma [2].

Here, we present two cases of metastatic bladder tumor from a primary gastric adenocarcinoma and a literature review of similar case reports.

## 2. Case Presentation

### 2.1. Case 1

A 72-year-old male patient with a history of smoking and no occupational exposure to urothelial carcinogens, was recently diagnosed with a poorly differentiating signet ring cell (SRC) carcinoma of the stomach based on a histological examination of endoscopic biopsy material taken from a suspicious infiltrating gastric mass.

Other than main gastric complaints, he complained of lower urinary tract storage symptoms such as frequency, nocturia, and urgency, but no haematuria was reported.

**Figure 1:**
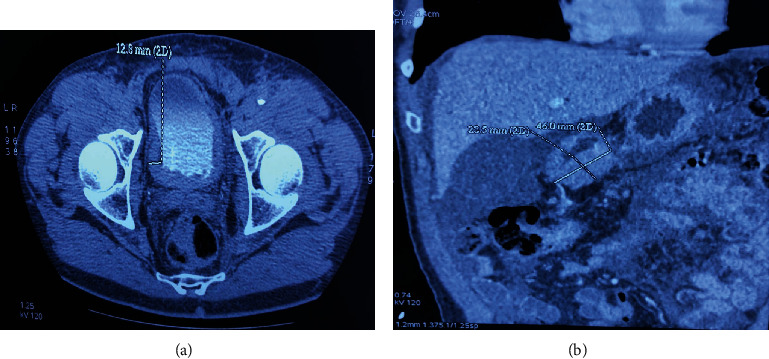
(a) CT scan showing diffuse irregular thickening of the bladder wall. (b) CT scan showing a neoplastic gastric mass.

A CT scan of the abdomen and the pelvis was performed showing the malignant tumoral mass in the pylorus with peritoneal involvement and ascites associated to a diffuse thickening of the bladder wall with bilateral hydronephrosis (Figure 1).

Physical pelvic examination was normal.

**Figure 2:**
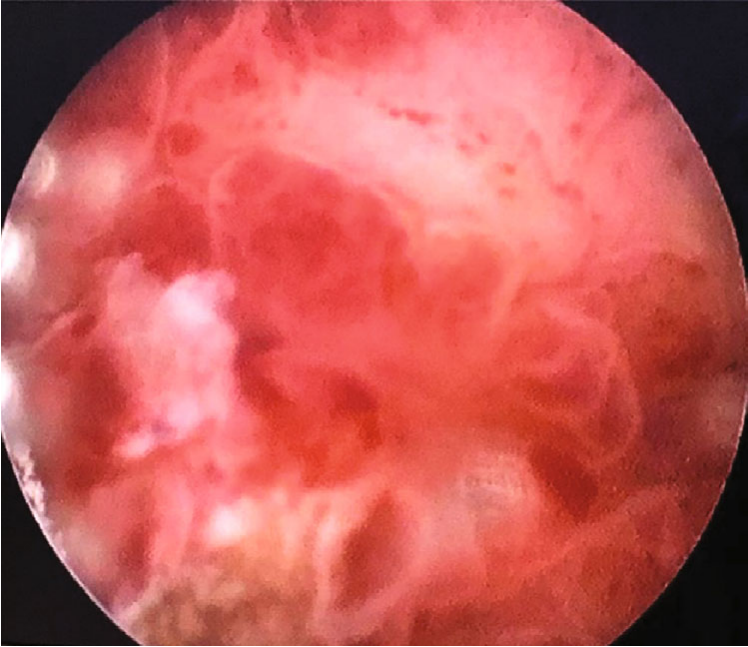
Macroscopic aspect of a bullous lesion of the bladder with a grape-like aspect.

Cystoscopy was performed, revealing an extensive bullous oedema of the bladder mucosa with a grape-like aspect with involvement of both the ureteric orifices initially not visualized, obscured by the lesion (Figure 2).

Transurethral biopsy of the lesion was conducted with liberation of the ureteric orifices.

**Figure 3:**
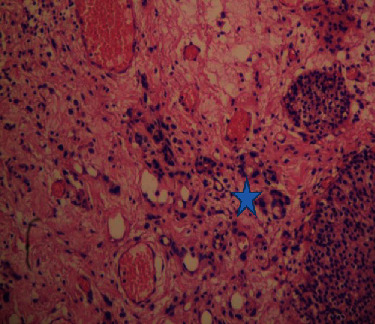
Infiltration of the subepithelium of the bladder with a small aggregates of neoplastic cells (H&E, ×200).

Histopathological evaluation of the bladder biopsy revealed multiple signet ring cells in the lamina propria with overlying transitional cell epithelium with no sign of urothelial carcinoma (Figure 3).

Based on histological findings and previous diagnosis of poorly differentiated gastric carcinoma, a diagnosis of synchronous bladder metastasis from primary gastric SRC carcinoma was made.

### 2.2. Case 2

A nonsmoker 36-year-old male with no occupational exposure to urothelial carcinogens, initially diagnosed five months ago with gastric signet ring cell adenocarcinoma metastatic to the bone with peritoneal involvement, initially treated with palliative chemotherapy, was referred to our urology department after he developed gross intermittent haematuria.

A CT scan of the abdomen and the pelvis detected two suspicious masses in the bladder wall, respectively, measuring 6 and 8 mm associated with homolateral hydronephrosis.

Because of our patient's history of primary gastric carcinoma, a secondary location of the bladder was suggested.

A cystoscopy showed two solid protuberant nodular lesions one in the trigone and the other in the right lateral bladder wall completely resected.

**Figure 4:**
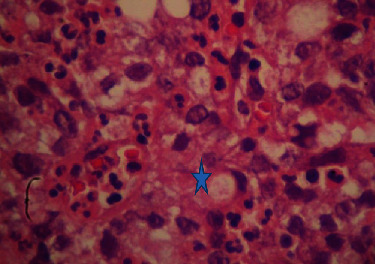
Signet-ring cells in the poorly differentiated adenocarcinoma (H&E, ×400).

After pathological examination of the surgical specimen, the diagnosis of metastatic poorly differentiated adenocarcinoma of the bladder from a gastric primary cancer was reached (Figure 4).

Both patients were then referred to oncology for a second-line chemotherapy.

## 3. Discussion

Bladder cancer (BC) is ranked the second most common genitourinary malignancy with an estimated 81000 new cases in the USA alone per year.

However, secondary neoplasms of the urinary bladder are rarely encountered in urology, accounting for only 2% of total vesical tumors according to the 2016 WHO classification [1].

Adenocarcinomas are the most frequent histological subtypes of such metastatic tumors [2].

Metastatic spread generally occurs by haematogenous or lymphogenous paths from distant primary foci.

Even intraperitoneal dissemination should be considered.

Bates et al. found that the most common sites of origin of cancer metastatic to the urinary bladder were the stomach yielding a figure of 4.3% of all secondary bladder neoplasms in a series of 282 cases [2].

After analysing 1000 consecutive postmortem cases of epithelial malignancies, Abrams' study yielded the same finding that gastric adenocarcinomas metastasize to the bladder more often than any other epithelial neoplasms [3].

We performed a review of PubMed for full-text peer-reviewed similar case reports published in English language since 1997.

We should point out that the majority of case reports occurred in Japan due to the high incidence of gastric adenocarcinoma there but were not included in this review due to the non-availability in English language.

15 case reports including our case report, involving 18 patients with secondary bladder tumor from primary gastric neoplasm, were included and analysed.

The features of these cases were summarised in Table 1.

The age range was 30-90, median of 60 years old.

Metastasis to the urinary bladder can be synchronous or can occur after the primary gastric tumor with an average of six years in our review.

Clinical urologic manifestations were absent in the majority of patients in whom bladder involvement was discovered postmortem [4]. However, in case one, our patient presented with lower urinary tract storage symptoms, and in case two, macroscopic haematuria was the chief complaint.

In our review, haematuria was the only consistent urinary symptom occurring in twelve of the total 18 patients.

Radiographic appearance of secondary bladder tumors may consist of focal or diffuse thickening of the bladder wall associated or not with hydronephrosis [5].

Distinction between metastatic bladder lesion and primary tumor may be hard from an imaging viewpoint which makes cystoscopy and histological examination the gold standard in making the diagnosis.

The metastatic bladder tumor can be described macroscopically as diffuse or protuberant similar to typical transitional cell carcinoma [6].

Most of the cases are protuberant in our review.

The main subsites of the secondary tumors were summarized in Table 2.

Of the 12 cases for which histology subtype was available, three were tubular type adenocarcinoma, and nine were signet ring cell type, whereas in Bates et al. series, out of the 10 cases, only three were signet ring cell [2].

Without clinical history of primary malignancy elsewhere, it is often challenging to distinguish between metastatic adenocarcinoma from primary adenocarcinoma, since primary bladder adenocarcinomas have a better prognosis following cystectomy [2], making immunohistochemistry necessary in that case .

Like other metastatic tumors, secondary bladder neoplasms have a variable chemosensitivity and radiosensitivity that correlates with the primary tumor [2].

Curative intention is not possible due to the metastatic characteristic of the disease and adjuvant chemotherapy is indicated, though with unsatisfactory results [4].

## 4. Conclusion

Despite being a rare disease, secondary bladder neoplasm should be considered when assessing a bladder lesion whether it occurs simultaneously or years after initial diagnosis of primary cancer.

Knowledge of its clinical and radiological characteristics is important for clinicians for correct diagnosis and proper therapeutic conduct.

## Figures and Tables

**Table 1:** Review of urinary bladder metastasis from a primary gastric tumor reported in English language literature.

Author and year	Age	Gender	Primary gastric tumor stage	Clinical presentation	Haematuria	Interval between primary tumor and metastasis	Imaging findings	Presence of hydronephrosis	Other metastasis	Endoscopic findings	Management of bladder metastasis	Follow-up
Saba et al. 1997 [7]	58	M	SRC AC	Nil	Yes	7 years HC	Polypoid lesion in the bladder posterior wall	No	Retroperitoneal adenopathy	Large broad-based lesion at the posterior wall, with mucinous strands	Nil	Deceased days after diagnosis
Kim et al. 2000 [4]	60	M	AC	Sense of residual urine dysuria, non-tender supra-pubic mass	Microscopic	One year HC	Focal thickening of the bladder anterior wall	No	Nil	NR	TUR	NR
Kim et al. 2000 [4]	57	F	SRC AC	Frequency, dysuria	Microscopic	Fifteen months HC	Diffuse bladder wall thickening	No	Nil	Bullous oedema of the bladder mucosa	TUR	NR
Kim et al. 2000 [4]	42	M	SRC AC	Dysuria	Microscopic	Two years HC	Diffuse thickening of the bladder wall	No	Nil	Oedematous mucosa of the bladder with a small ulcer	Total cystectomy	NR
Antunes et al. 2004 [8]	63	F	AC	Left lumbar pain, dysuria low abdominal pain	Nil	One year and nine months	Thickened wall of the bladder	Bilateral	Ascites	Extensive vegetative lesion in the bladder	TUR	Stable condition 8 months after diagnosis
Matsuhashi et al. 2005 [9]	90	F	Tubular AC	Nausea, dysphagia	Ye	Synchronous metastasis	Thickening of the bladder wall with diverticulum enhance effect	No	Nil	Lesion in the bladder diverticulum	NIL	Deceased three months after diagnosis
Farhat et al. 2007 [10]	58	M	AC	Nil	Yes	Fifteen months HC	2 cm mass involving the trigone and the left lateral bladder wall	No	Nil	NR	TUR	NR
Sharma et al. 2011 [6]	30	M	SRC AC	Weight loss	Yes	Two years HC	Localized thickening of the bladder wall	No	Nil	Multiple grape-like lesions on the dome and left bladder	TUR adjuvant chemotherapy	Alive five months after chemo
Andras et al. 2013 [11]	59	M	Tubular AC	Low abdominal pain	Nil	Ten years HC	2 × 4 cm tumor-like mass on the left posterior bladder wall	No	Nil	Mucosal hyperaemia lesion close to the ureteric orifice	TUR adjuvant chemotherapy	Colic recurrence one year after chemo
Kalra et al. 2015 [12]	60	M	SRC AC	Low urinary tract storage symptoms	Microscopic	Synchronous metastasis	Diffusely thickened bladder wall with small capacity bladder	Bilateral	Nil	Smooth wall diffusely erythematous small capacity bladder	TUR nephrostomy diversion Palliative chemotherapy	
Okutur et al. 2015 [13]	48	M	SRC AC	Abdominal pain, weight loss tenderness of hypogastric region	Yes	Synchronous metastasis	Diffuse thickening of the bladder wall	Bilateral	Peritoneum	Diffuse papillary nodular lesion	Transurethral biopsy Palliative chemotherapy	Alive 5 months after chemotherapy
Lodh et al. 2016 [14]	53	M	Tubular AC	Abdominal fullness weight loss	Yes	Synchronous metastasis	Right posterior lateral urinary bladder broad base mass	No	Nil	Protuberant mass	TUR adjuvant chemotherapy	Stable three month later
Vigliar et al. 2013 [15]	38	M	SRC AC	Abdominal pain	Yes	Seven months HC	NR	Unilateral	Ascites	NR	NIL	Deceased nine months after diagnosis
Seow-En et al. 2015 [16]	75	M	SRC AC	Frequency	Nil	Twenty years HC	Bladder wall thickening	Bilateral	Lymphadenopathy rectum	Mucosal tumor growth	Chemotherapy	Alive four months since diagnosis
Khoury et al. 2019 [17]	75	M	SRC AC	Appetite loss, lumbar pain	Nil	Two years	Diffuse thickening of bladder wall	Bilateral	NIL	Extensive vegetative lesion	NR	NR
Ota et al. 1999 [18]	43	M	AC	Sense of residual urine, incontinence	Nil	Two years HC	Diffuse thickening of bladder wall	Bilateral	Nil	NR	Chemotherapy	Alive twelve months after chemotherapy

SRC: signet ring cell; AC: adenocarcinoma; TUR: transurethral resection; HC: heterochronous; NR: not reported.

**Table 2:** Anatomical localization of secondary tumor deposits within the urinary bladder of the reviewed cases.

Neck	Trigone	Anterior/lateral/posterior	Fundus	Diffuse
0	3	7	0	4

## Data Availability

Data availability is accessible on demand.
